# A systematic review of health communication strategies in Sub-Saharan Africa-2015-2022

**DOI:** 10.34172/hpp.2023.02

**Published:** 2023-04-30

**Authors:** Adewale Olaoye, Kevin Onyenankeya

**Affiliations:** University of Fort Hare, Alice, Eastern Cape, South Africa

**Keywords:** Communication, Health, Promotions of health, Sub-Saharan Africa, Review, Systematic

## Abstract

**Background:** Health communication strategies have become critical in managing public health issues across sub-Saharan Africa. In the literature, health communication strategies have been well documented. The studies are often narrow, focusing on individual countries or specific health issues. No research documented and consolidated the health communication strategies across sub-Saharan Africa. This review attempts to catalogue prevalent health communication strategies, how the various countries have implemented these strategies and the barriers to effective health communication practices in Africa.

**Methods:** We systematically reviewed existing literature on health communication strategies in sub-Saharan Africa to answer formulated questions. A Google search was performed in October 2022 with the keywords ‘health communication’, ‘strategies’, ‘promotion,’ ‘education,’ and ‘engagement,’ The data reported in this article included evidence published between 2013 and 2023. Selected documents were content analyzed, and significant sections were mapped against specific strategies/themes. These subsets of data were used to present the results and analysis.

**Results:**The review indicates that different health communication strategies have been deployed across Africa. In some countries, specific strategies are used to tackle specific health issues, while a combination of strategies is used in others. In some countries, the strategies are unclear, and implementation is improvised, sometimes misapplied, or truncated by bureaucratic red tape and incompetence. The prevalent strategies are mainly those prescribed from outside with little input from the beneficiaries.

**Conclusion:** The review suggests that using a holistic or multi-pronged health communication approach that is context-specific and participatory could attract more uptakes of health messages.

## Introduction

 Health communication is vital to the health ecosystem, especially in managing public health crises and promoting healthy living.^1-4^ Health communication refers to using communication theory, evidence, techniques, and creativity to inform, educate and influence public views and perceptions and promote behaviours and practices that advance the health and wellbeing of individuals and communities.^3^ Although the design, delivery and theoretical foundations of health communication have evolved over the past decades shifting significantly from ‘text/broadcast-based to digital messaging and social media,^5^ the overarching goals of health communication remain to increase risk perception, reinforce positive actions, influence social norms, and change individual or group attitudes and behaviours.^4^

 In the literature, health communication approaches revolve around community engagement, risk and crisis communication.^6^ Risk communication involves conveying likely risks to public health, engaging those who might be at risk on prescribing actions to avoid the potential threat.^7^ On the other hand, crisis communication addresses a current crisis or public health issue that just happened. The two strategies are somehow intertwined as risk can trigger a crisis.^8^ and can be used simultaneously as an integrative campaign.^9^

 Health communication strategies are critical for the African continent faced with a slew of infectious-disease epidemics, pandemics, and chronic diseases.^10,11^ The recurrent epidemics negatively impact the already weak healthcare systems, devastate the struggling economies, and lead to a colossal toll on human life.^12^ Managing the multifaceted health challenges, especially public health emergencies and the attendant physical, psychological, and socioeconomic impact, requires developing and applying well-thought-out and effective health communication strategies consistent with the population’s cultural, demographic, and psychographic peculiarities.

 There is a raft of health communication strategies to influence health behaviour or outcomes acrosssub-Saharan Africa. The common strategies include information, education and communication (IEC)^6,12-20^leveraging the mass media^21-27^ and social media/ICT^28-39^ Other strategies include communication campaigns or social marketing^40-47^ entertainment education^48-50^ involving theatre,^51-58^ film,^59,60^ music and songs.^61-68^ Interpersonal communication^67-69^ that employs community and religious leaders^13,69-78^ and community engagement.^79-89^

 Although the strategies vary, the common purpose is to inform, educate and communicate potential health risks and manage public health emergencies.^90^ In many African countries, health communication strategies lack coherence and crisis and risk communication strategies need to be better developed.^91,92^ The strategies are poorly implemented due to inadequate funding,^93^ lack of capacity and red-tapism. Most risk communication strategies applied in sub-Saharan Africa are externally funded,^8,9,42,94-96^ especially art-based health promotion campaigns, adopted from ‘western, educated, industrialized, rich democratic contexts.^64,91^ The seemingly Western-themed-one-size-fits-all interventions overlook the recipients’ sociocultural and religious dynamics,^8,64,94,95,97-99^ which compromises health information uptake and participation in health education programmes. This is not peculiar to sub-Saharan Africa. In Iran, Hamidzadeh et al^100^ found several obstacles impede health information promotion and community participation in rural health education programmes, including a lack of understanding of the importance of health education. Despite its weaknesses, health communication in sub-Saharan Africa has proved essential in providing meaningful information and cultivating attitudinal and behavioural change among people regarding personal and public health issues. However, some scholars^101-103^ have made a case for a culturally nuanced agenda in articulating African health communication strategies.

 However, only a few low- and medium-income countries have ‘one-stop shops’ where they can readily access information regarding health policy and health systems.^104^ There is scanty information on health communication and its effectiveness in Africa. Except for campaigns on birth rate reduction and child survival, available evidence on health communication effectiveness is generated from high-income countries in the global North and Asia, where most campaigns are implemented, and there is substantially greater research capacity.^105^

 The few research that has examined African health communication strategies are often narrow, concentrating on individual countries, regions, specific health issues or media tactics. No research documented and consolidated the gamut of health communication strategies across Africa. This review attempts to document and consolidate the general health communication strategies in sub-Saharan Africa and highlight how the various approaches have been applied in specific health intervention efforts. The review’s goal is not to comprehensively examine the individual country’s health communication campaigns or to undertake a Meta-analysis or statistical summary of the individual studies but to sketch and aggregate strategies common across the continent for easy reference among health communication researchers. The overarching questions in this review are 1. What are the prevalent health communication strategies in Africa? 2. What are the underlying communication issues and concerns?

## Material and Methods

 The reporting of this systematic review was guided by the standards of the Preferred Reporting Items for Systematic Review and Meta-Analysis 2020 (PRISMA) Statement.^106^

###  Eligibility criteria

 The search identified recent literature on health communication strategies in sub-Saharan Africa, including grey literature published between 2013 and 2022. Documents included in the review must focus on health communication strategies, including crisis and risk communication to eradicate or combat the spread of infectious diseases, epidemics, pandemics and other public health issues. We excluded documents dealing with health systems and policies unrelated to health communication to educate and change behaviour.

###  Information sources

 A pilot scan of grey literature in the PDQ-Evidence repository, Health Technology Assessment Database, the Virtual Health Library, greylit.org and worldcat.org yielded scanty entries. The search was then limited to scientific publications in PubMed and Google Scholar.

###  Search strategy

 A Google search was performed in October 2022 with the keywords “health communication”, ‘strategies’”, “promotion”, “education,” “engagement”, and “Africa”, yielding 3620 after overlapping documents were filtered.

###  Selection process

 Selected articles must contain strategies aimed at (1) educating the population about the causes of and preventive measures, (2) creating awareness about symptoms and treatment, (3) tackling misconceptions and myths, (4) calling to action, and (5) must focus on Africa. Additionally, the scanned articles must be consistent with clearly defined research questions.

###  Data collection process

 Data were sought from the electronic databases for five domains – IEC, health campaigns or social marketing, interpersonal communication, entertainment education (E-E) and community involvement. The search yielded 3620 documents, of which 86 met the inclusion criteria. The total number of scanned documents is an estimate and not a representation of individual, separate documents, as many of the papers overlapped, with the same articles appearing multiple times in the results returned. Two reviewers (AO & KO) extracted key documents onto a standard template.

###  Study risk of bias assessment

 The risk of bias assessment was mitigated by selecting only items that conform to the clearly defined inclusion and exclusion criteria.

###  Synthesis method

 Selected documents were content analyzed, and significant sections were mapped against specific strategies/themes. A file was created for each strategy/theme, and relevant information and sources were assigned accordingly. These subsets of data were used to synthesize and present the results.

## Results

 Of the 86 documents reviewed, 96% were empirical studies and grey literature (4%). Most documents focused on health communication strategies in Western Africa (45%), Southern Africa (25%), and East Africa (15%). Of the 86 included in this review, 46% reported on risk communication in response to and reducing the risk of transmitting and spreading the coronavirus in African countries.^6^ The bulk of the documents focused on strategies addressing infectious diseases such as COVID-19, Ebola, HIV/AIDS, malaria, pneumonia, and TB, which are the major health problems in the period under review. Only two documents focused on non-communicable diseases caused by unhealthy lifestyles such as smoking, harmful alcohol consumption, unhealthy diets, and sedentary or physical inactivity. It was evident from the review that stakeholders see health communication as a vital and valuable tool in highlighting public health risks and promoting healthy living.^38^ Many documents highlighted the need for a culturally nuanced and participatory approach in designing and delivering health communication strategies in the African continent.^8,67,93,101-103^

 In ascending order, sub-Saharan Africa’s prevalent health communication strategies can be classified into five broad categories – IEC, health campaigns or social marketing, interpersonal communication, E-E and community involvement (Table 1). The results are presented along these themes.

**Table 1:** Prevalent health communication strategies in Africa

**Health communication strategies**	**Targets**	**Vehicles**
Information education and communication (IEC)	Mass audience	Radio, TV, social media
Health communication campaigns or social marketing campaigns	Mass/targeted audience	Media, social media
Entertainment education	Mass audience	Radio/TV drama, celebrities/ stories/songs
Interpersonal communication (IPC)	Individuals/small groups	Individuals, health workers
Community engagement	Groups/community	Grassroots movements/ networks/church/community leaders

###  Information, education and communication (IEC)

 IEC is a deliberate and planned public health technique where messages or information are disseminated to individuals or target audiences using appropriate communication channels to create awareness, educate and change knowledge, belief, attitude, and behaviour.^107,108^ The review found IEC approach is the most prevalent health communication strategy across African countries. Health departments often drive the IEC strategy and target a specific problem. IEC is primarily deployed to inform people about health illnesses and ways to protect themselves or to change or reinforce health-related behaviour. A second use of IEC is risk communication to break the news about health risks and create awareness during emergencies.^12,109,110^ This method was used extensively to remove myths and misconceptions and encourage people to adopt and maintain more healthful practices during the COVID-19,^6^ and Ebola outbreaks in West Africa.^12,13^

 The IEC strategy is also used to prevent high-risk behaviour^14-16^ and other lifestyle-related diseases such as obesity^16^ and promote positive behaviours, including vaccination, physical exercises and seeking healthcare services.^17,18^ IEC was prevalent in the fight against HIV/AIDS, especially in disproving myths and misinformation about the disease and discouraging discrimination and stigmatization of victims.

 In Rwanda, one document report that tailored IEC is used to inform and educate rural and urban populations about cardiovascular diseases encouraging risk screening and lifestyle modification critical in combating the disease, the country’s third leading cause of death.^17^ In Kenya, IEC is crucial in promoting parent-child sexual and reproductive health communication. A document that investigated the uptake of sexual and reproductive health communication among very young adolescents in Nairobi, Kenya, found that parents can be important, reliable sources of sexual and reproductive information and significantly influence adolescents’ sexual values, attitudes, and risk-related beliefs.^16^ Similarly, in Malawi, IEC is the base of the national health communication strategy covering malaria, HIV, Maternal, nutrition and tropical diseases.^20^

 To be effective, IEC must use a range of communication channels, as most IEC materials alone cannot muster the desired behaviour change.^107^ However, due to insufficient resources, most African health departments and governments limit their IEC endeavours to publicity, information or awareness creation leaving out health education and activities that should motivate attitudinal and behavioural change. The review showed that various communication channels are used to execute the IEC communication across the continent. They include the mass media (radio, television, social media, out-of-home), interpersonal (face-to-face conversation, counselling, home visits), Community/group (community heal workers. discussions, lectures/workshops, demonstration, or role-play, outreach), Printed materials (flyers, leaflets, brochures). The following section discusses how various countries have applied these channels to implement their IEC strategies.

###  Mass media 

 The review indicates that the mass media, radio, television, and newspaper are the primary communication channels used for health communication. Two documents^12,19^ indicated that public service announcements (PSAs) were a common mass media vehicle and could effectively create awareness about social and health issues and influence a change of attitude and behaviour toward such issues. Radio PSA, was a key channel for IEC messaging around COVID-19 risk communication interventions and COVID-19 vaccination drive, polio immunization campaigns, Ebola prevention education campaigns and the fight against mental health illness, especially the stigmatization of victims.^12,21,23,24^

 Yaya and Bishwajit^29^ explored how family planning communication disseminated through radio and television promote maternal healthcare utilization in Nigeria. Similarly, Ethiopia used radio and television, to execute its IEC interventions around cholera.^26^ However, media advocacy as a strategy for public health communication produced relatively low documents. One document reports that national and regional governments used media advocacy to create awareness and tackle the myth and misconceptions about sickle cell disorder.^27^

###  Social media/ICT 

 The result indicated a growing interest in social media/ICT use to inform, educate, and communicate health information across sub-Saharan Africa. Three documents reported that social media and ICT have proved to be effective communication tools for delivering IEC in African countries.^28-30^ One document holds that social media presents a significant opportunity to enhance programmes and campaigns and support public health initiatives,^31^ especially when combined with other communication strategies.

 The prevalent social media and ICT platforms used are Facebook, YouTube, Twitter—WhatsApp, cell phones, and SMS, especially in crisis communication.^29^ Twitter was strategic in Nigeria’s Ebola information dissemination efforts, especially in providing accurate information and tackling the hoax surrounding the disease.^24^ Nigeria also used mobile phones extensively to disseminate SMS during the Ebola outbreak^28,29^ and COVID-19 communication.^6^ In Sierra-Leon, SMS was used for real-time monitoring.^32^ while WhatsApp messaging helped to dispel Ebola rumours and myths and communicate with people quarantined during the crisis.^30,33^

 Kubheka et al^31^ reported social media’s use in South Africa’s health promotion, including the *Haybo wena* TB campaign, which leveraged technology cell phones to reach and engage the younger generation.^34,35^ In Malawi, Malanga^36^ reports on mobile health initiatives such as the *Chipatala Cha Pa Foni* and the Rapid SMS projects, which deployed ICT to deliver messages to improve maternal, newborn and child health. Mobile phones also facilitated the delivery of telemedicine or mobile health to people in poorly resourced settings in Botswana.^37^ The practice is also evident in South Africa, especially among private health providers. The mobile health messaging service, *MomConnect* uses platforms such as WhatsApp, SMS, and *MXit* to provide information and support to pregnant women.^38^

 The results revealed that virtually all the national health ministries have functional websites or information portals providing various health-related information. One document noted that although social media offers tremendous opportunities for health communication, it poses some challenges, one being its susceptibility to being used to purvey misinformation.^29^ Furthermore, social media platforms such as Facebook, YouTube, and Twitter focus on urban areas isolating rural dwellers.^30,39^ While science and technology is important in combating public health issues and emergencies, direct human participation is still crucial, as demonstrated by the Ebola and COVID-19 outbreaks. Social mobilization in the form of face-to-face discussions and folk theatre is as important as technologies and innovations in health communication delivery.^39,95^

###  Communication campaign or social marketing strategy 

 A communication campaign refers to a “purposive attempt to inform or influence behaviours in large audiences within a specified period using an organized set of communication activities and featuring an array of mediated messages in multiple channels generally to produce noncommercial benefits to individuals and society.”^40^ The review found that although health communication campaigns are prevalent across African countries, home-grown interventions are lacking and poorly funded. Most health campaign initiatives are ‘pre-packaged’ from the outside or adapted from multilateral organizations, especially theatre-based initiatives.^93,97^ The campaigns generally target specific health issues to achieve specific outcomes or effects, such as combating the spread and impact of the HIV/AIDS epidemic,^8^ cigarette smoking, teenage pregnancy, reproductive health,^17^ physical exercises and healthy dieting at mitigating cardiovascular diseases.^16^

 Some well-resourced national health ministries, like the South African health department, use advertising in their health communication campaigns. The *Haybo wena* TB campaign employed a social marketing strategy combining media channels, including ICT and below-the-line advertising materials such as T-shirts, pamphlets to create awareness and education around TB.^34,35^

 Another social marketing strategy is theUSAID-sponsored* We Beat TB* Campaign incorporating animated TV slots and PSA that ran on public and private TV and radio stations, The *Phila Campaign*, broadcast on television and radio with the tagline *Inspired to Live.*^41,42^ The campaign provides health information regarding HIV, TB, non-communicable diseases and mother, child and women’s health. However, one document noted that running messages on two issues concurrently resulted in TB messages being overshadowed.^73^

 Social marketing has been a significant plank in promoting exclusive breastfeeding campaigns in many African countries, including Ghana and Niger where it was used to reach socio-economically vulnerable mothers and promote early breastfeeding initiation after birth in rural areas.^74^ The strategy was used in the *Baby-Friendly Hospital Initiative*, *Alive & Thrive Initiative* in Nigeria,^12,44^ the* side-by-side, Road-to health booklet* and *Mom connect* in South Africa.^45^

 While one document indicates that combining communication channels, such as mass media, social media, and IPC, could increase exclusive breastfeeding,^44^ others reported suboptimal knowledge about breastfeeding among mothers in East African countries.^47^ One explanation for this may be the intermittent nature of the campaigns. Providing a year-round communication campaign using a variety of communication channels and platforms could improve the efficacy of the exclusive breastfeeding campaigns.^45^

###  Entertainment education

 Entertainment education also known as edutainment, refers to “the intentional placement of educational content in entertainment messages”^48^ such as dance, drama, and pop culture to inform, educate, and improve a target population’s knowledge about a health issue and ultimately influence a change in attitude or behaviour towards the issue.^50,60^ Edutainment can be vital for attracting and involving underrepresented communities in stimulating individual and group interest in public health issues.^50^

 The result indicates that E-E has come to occupy an important position in health communication delivery strategies in many sub-Saharan Africa n countries. Govender^49^ attributes E-E’s popularity in health promotion in Africa to its affordance of a more innovative way to communicate public health emergencies and influence a desired attitudinal behavioural change.

 Consistent with a previous study,^51^ the review showed that multiple E-E or Arts-based strategies such as music, songs, theatre, film, and folk theatre are increasingly being deployed in promoting health issues in sub-Saharan Africa. E-E was prevalent in the HIV/AIDS and Ebola prevention, care, and support campaign.^49,51,52^ For instance, in South Africa the Soul City, a radio and teledrama series, *Sarafina 11 *musicalandtelevision Soapies such as *Isidingo*^53,54^ and the *Tsha-Tsha* was deployed to combat HIV/AIDS pandemic by engaging young people on HIV/AIDS issues, including HIV-related stigma, disclosure, condom use, secondary abstinence, voluntary counselling and testing, and sexual assault. The success of *Tsha-Tsha* was hinged on its novel cultural approach and use of lingua franca.^55^

 Another notable edutainment vehicle is the MTV drama series *Shuga* aired across many sub-Saharan countries. The drama promotes positive sexual behaviour and abstinence, discourages infidelity, transactional sex, and stigmatization of people living with HIV and addresses other issues confronting the youth, including teenage pregnancy, peer pressure, and identity issues.^56^*Shuga* effectively conveyed strong and consistent destigmatizing and condom-promoting messages in Kenya.^57^ In Rwanda, the *Impano n’ Impamba*radio drama helped to educate and engage listeners about pregnancy risk.^58^ Similarly, in Mozambique, the radio drama *Ouro Negro* (Black Gold) promotes personal health and behavioural change.^18^

 Film is also a critical E-E strategy. In Liberia, the film *Killer Bean* and *Falcao* proved important in preventing the spread of the Ebola virus and changing public perceptions about the disease.^59^ The *Majigi*film was used to combat the refusal of the oral polio vaccination in Northern Nigeria.^60^ the *Haybo wena* TB campaign played an essential role in reducing the spread of TB in South Africa.^34,35^

 Another popular edutainment strategy revealed in the review is music and songs, which were influential in creating awareness and communicating preventive measures during COVID-19^65,66^ and Ebola pandemic in Liberia. Nigeria, Gambia and Ghana^52,61,63,64^ and HIV/AIDS behaviour change in Ethiopia, Tanzania, and Uganda.^68^ The songs fostered trust in the messaging and provided reach that conventional methods would not have achieved.^52^ Music also effectively created awareness about the challenges and use of contraceptives to prevent unwanted pregnancies in Nigeria.^67^

###  Interpersonal /group communication

 In its most basic definition, IPC refers to exchanging verbal and nonverbal information between two or more people or intimate groups^69,70^ in face-to-face or social media settings.^71^ In IPC influential individuals use their interpersonal standing and skill to deliver health information and influence people under their sphere of influence.

 The results indicate that IPC strategies involve local groups and organizations, especially religious groups focusing on delivering health information. IPC and dialogue were used to boost exclusive breastfeeding practices among nursing mothers,^44,46^ dispel rumours about the Ebola virus,^44^ and create awareness about HIV/AIDS, promoting safer sexual practices and advocating a stop to discrimination and stigmatization of victims.^76^ Many countries are leveraging the political establishment and local groups such as faith-based leaders for interpersonal or face-to-face health promotion, especially in the HIV/AIDS campaign^75,76^ In South Africa IPC was deployed to mobilize and sensitize converts about the risks of attending burials^77^ and other religious and cultural aspects that had previously been overlooked.^78^

 The results showed that IPC is deployed to improve patient care in a patient-provider communication (PCC) setting such as nurses and doctors. While PCC practice has increased in sub-Saharan Africa,^4,72,73^ the implementation has been fraught with challenges. Across sub-Saharan Africa, participants perceived healthcare providers mostly as uncaring, unfriendly, and disrespectful.^74^ The results suggest that PCC had received scanty attention in the literature, and current debates on the issue appear to focus on HIV and AIDS, TB, and maternal health, suggesting a need for more patient-centred care research that focus on understanding PCC in various contexts.^74^

###  Community involvement

 Community involvement is one of the common health communication strategies in sub-Saharan Africa. Community involvement is often deployed in patient-public engagement (PPE) and can be implemented on five levels, including traditional leadership support.^79^ Community mobilization remains an essential strategy in cascading health information to the grassroots. The results show that community engagement was a critical strategy in controlling COVID-19 transmission in many African countries.^6,80^ In Ethiopia, health extension workers created awareness around COVID-19 in hard-to-reach areas.^82^ At the same time, religious leaders helped to sensitize and mobilize the public on COVID-19 safety measures in Uganda.^87^ Women groups and burial societies in Zimbabwe increased uptake in HIV testing, counselling, and prevention-of-mother-to-child transmission.^83^

 Social mobilization and community engagement were critical in managing the Ebola outbreak in Guinea and Sierra Leone^84^ and the fight against Ebola in Liberia.^85^ Community interventions proved to be a successful and viable means of promoting cardiovascular health in several countries despite the challenges in implementation.^86^ In Uganda, one document reports the impact of the *CHW* programme - the *village health teams* (VHTs), in promoting health issues, despite poor community support.^88^ Similarly, in Malawi, a local network *Juma*Community Health Action Group, played a pivotal role in combating the Malaria scourge by using drama, songs, and poetry to create awareness about Malaria prevention and treatment.^15^ In Mozambique, two local networks - *h2n* and *Tchova-Tchova Association for Community Programs* were crucial in the fight against malaria. One document indicates that the involvement of civil society groups and community leaders was significant in promoting optimal breastfeeding practices in Nigeria and South Africa.^44^

 However, community involvement, especially PPE, appears symbolic, as little community participation occurs in the implementation stages, which are currently concentrated at the ‘service design’ or health research levels.^79^ Limited community representation, lack of administrative support, capacity building, and policy commitment compromise community involvement in health promotion initiatives.^89^ Innovative PPE approaches that incorporate communities’ economic development could mitigate the implementation challenges^86^ leading to effective and enduring outcomes.

## Discussion

 The review suggests that although many African countries have an elaborate national health plan, only a few countries, such as South Africa, Ghana, Nigeria and Malawi, have clearly defined standardized and curated national health promotion or communication strategies embedded in national health plans. While most African health communication strategies have lofty objectives, the implementation has been haphazard in some countries and shoddy in others. This is due mainly to a lack of capacity and inconsistency. There is a sense that the definition and practices of health promotion in Africa are not well understood and documented.^92^ The health communication strategies focus on providing education and information about the numerous health challenges - reproductive health, child and maternal health and nutritional deficiencies and galvanizing the populace in the face of public health emergencies such as outbreaks, epidemics and pandemics or combating the spread of infectious diseases.^90^ Fewer campaigns address lifestyle-induced diseases such as diabetes, cardiovascular diseases, and obesity. Little or no health campaigns focus on mental health, which is increasingly becoming a public health concern and continues to attract little scholarship and funding across the continent.^111^

 The strategies deployed vary across the continent. In some countries, specific strategies are used to tackle specific health issues, while a combination of health communication strategies is used in others. In many countries, the strategies are unclear, and implementation is improvised, sometimes misapplied, or truncated by bureaucratic red tape and incompetence. Many lack well-developed and coherent crisis and risk communication strategies. This aligns with previous studies that found many sub-Saharan Africa n countries lack well-developed crisis and risk communication strategies.^91^

 The underlying communication issues and concerns emerging from the review is that the preponderance of existing health communication strategies in Africa is adopted from the Global South^99^ and grounded on Western ideologies, especially crisis communication messaging. Consistent with previous studies, the result shows that most health communication designs in content and delivery, have subdued local involvement.^36,37^ The fact that Western donor agencies fund most public health promotion activities^8,96^ is a plausible reason for the prevalence of Western-oriented ideologies in African health information messaging. Multilateral agencies and partners such as WHO, UNICEF, UNDP, UNESCO, USAID, World Bank, and John Hopkins produce most social marketing campaigns across sub-Saharan Africa.^8,42^ Using ready-made strategies and health communication messaging that do not sufficiently address sociocultural variables^97^ leading to conflict with local cultural and religious values^8,98^ and poor uptake of health information.

 When the strategies are produced locally, the foreign sponsors often prescribe or micromanage the production and final content.^8^ This tendency inevitably leads to applying one-size-fits-all strategies, which research has shown is not applicable in many contexts.^110^ The success or failure of any health communication effort depends on how well the strategy is conceived and how well contextual elements, including cultural nuances, linguistic diversities, and ethnic peculiarities, are incorporated in the design and execution of the strategy. Many African countries faced sociocultural and religious resistance to HIV/AIDS and COVID-19 messaging because the risk communication strategies did not sufficiently reflect sociocultural variables.^18,28^

 This highlights the need for a culturally nuanced African health communication strategies. Harnessing and combining Global North’s communication strategies^94,101,102^ with traditional approaches incorporating indigenous knowledge systems and community groups could beneficial and mitigate resistance to health information.^64^ As demonstrated in the literature, anchoring health communication messages, stories, and drama in indigenous languages can also help build trust with the community and improve the chances of health information uptake.^103^

## Conclusion

 This paper reviewed health communication strategies in sub-Saharan Africa to ascertain the general approaches and highlight underlying communication issues and concerns regarding their implementation. It was evident that health communication strategies have become critical and frontal in managing public health issues across Africa. IEC, Social marketing, E-E, IPC and Community engagement emerged as the predominant health communication strategies in the continent. Although there appears to be no ‘one size fits all’ health communication strategy, most countries prioritize IEC and E-E strategies based on social learning theory.

 The review indicates that although health initiatives are driven by the national health departments and local organizations, the preponderance of health communication interventions and messaging are designed and funded by donor agencies, which tend to limit the participation of those the communication was intended to reach. The dependence on externally prescribed strategies often leads to the muffling or outright neglect of local sociocultural and religious undercurrents. The review suggests a need for more traditional health communication strategies based on indigenous knowledge systems. The review concludes that using a holistic or multi-pronged health communication approach that is context-specific and participatory could attract more uptakes of health messages.

## Competing Interests

 None.

## Ethical Approval

 Not applicable.
